# Immunotherapy with Cell-Based Biological Drugs to Cure HIV-1 Infection

**DOI:** 10.3390/cells11010077

**Published:** 2021-12-28

**Authors:** Gabriel Siracusano, Lucia Lopalco

**Affiliations:** Division of Immunology, Transplantation and Infectious Diseases, San Raffaele Scientific Institute, 20128 Milan, Italy

**Keywords:** CCR5, TLR, antibodies, neutralizing antibodies, HIV blocking antibodies, CD4bs, CD4i

## Abstract

Since its discovery 35 years ago, there have been no therapeutic interventions shown to enable full HIV-1 remission. Combined antiretroviral therapy (cART) has achieved the sustained control of HIV-1 replication, however, the life-long treatment does not eradicate long-lived latently infected reservoirs and can result in multiple side effects including the development of multidrug-resistant escape mutants. Antibody-based treatments have emerged as alternative approaches for a HIV-1 cure. Here, we will review clinical advances in coreceptor-targeting antibodies, with respect to anti-CCR5 antibodies in particular, which are currently being generated to target the early stages of infection. Among the Env-specific antibodies widely accepted as relevant in cure strategies, the potential role of those targeting CD4-induced (CD4i) epitopes of the CD4-binding site (CD4bs) in eliminating HIV-1 infected cells has gained increasing interest and will be presented. Together, with approaches targeting the HIV-1 replication cycle, we will discuss the strategies aimed at boosting and modulating specific HIV-1 immune responses, highlighting the harnessing of TLR agonists for their dual role as latency reverting agents (LRAs) and immune-modulatory compounds. The synergistic combinations of different approaches have shown promising results to ultimately enable a HIV-1 cure.

## 1. Introduction

**Figure 1:**
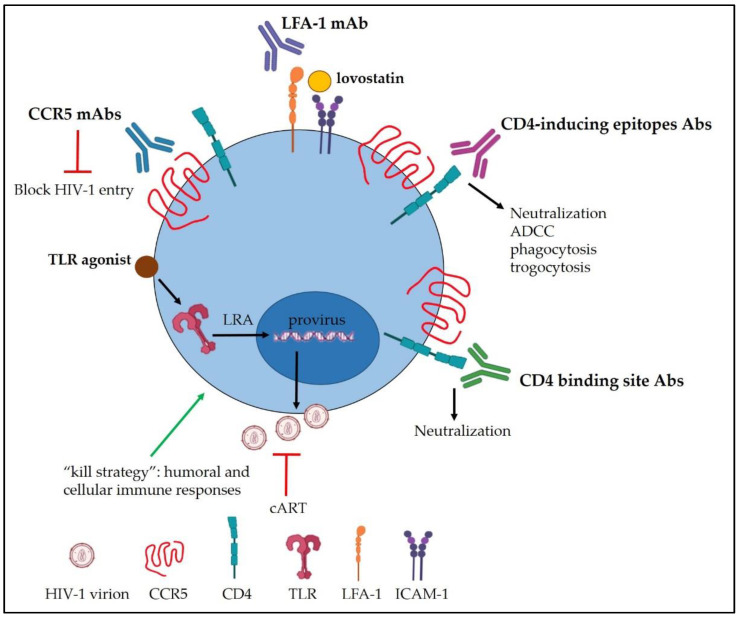
Graphical sketch of current approaches for cell-based immunotherapy.

At the end of December 2020, 27.5 million people were accessing antiretroviral therapy (ART), up from 7.8 million in 2010. This life-long treatment reduced new human immunodeficiency virus type 1 (HIV-1) infections by 52% since its peak in 1997 [1], as well as the progression to Acquired Immunodeficiency Syndrome (AIDS) and the complications associated with inflammation [2]. When combined, anti-retroviral drugs provide complete viral suppression for several years. Challenges to the global eradication of HIV-1 infection include the persistence of viral reservoirs of latently infected cells, mainly resting CD4+ T lymphocytes, in which the virus reactivates once the therapy is interrupted [3,4]. Moreover, HIV-1 infected patients experience non-AIDS diseases including cardiovascular and bone diseases and cognitive disorders [5]. To overcome these barriers, new strategies have been implemented to achieve a more effective curative intervention to eradicate HIV-1. Here, we will review the preclinical and clinical advances of cell-based immunotherapies. We will focus on coreceptor-targeting antibodies (Abs), particularly those targeting CCR5, and among the Env-specific antibodies, we will point out those targeting CD4-induced (CD4i) epitopes of the CD4-binding site (CD4bs). Together with approaches targeting the HIV-1 replication cycle, we will discuss the strategies aimed at boosting and modulating specific HIV-1 immune responses, highlighting the harnessing of Toll-like receptors (TLR) agonists for their dual role as latency reverting agents (LRAs) and immune-modulatory compounds. A schematic overview of the approaches discussed here is reported in Figure 1. Ninety clinical trials based on the targets reported in this review are registered on ClinicalTrial.gov (accessed on 24 November 2020).

## 2. Anti-CCR5 Antibodies

HIV-1 utilizes either the CC chemokine receptor 5 (CCR5) or the C-X-C Motif Chemokine Receptor 4 (CXCR4) to enter into CD4^+^ cells [6]. However, CCR5, a chemokine receptor belonging to the G protein-coupled receptors (GPCRs) superfamily, is the principal HIV-1 co-receptor, involved in virus entry and cell-to-cell spread [7] in addition to its main role in mediating the activation and migration of T lymphocytes [8]. Subjects with a homozygous 32 base pair deletion (Δ32) in the ccr5 gene, causing a frameshift and premature stop codon that encodes for a truncated form of the protein that is not expressed on the cell surface, are highly resistant to HIV-1 infection [9,10]. Natural anti-CCR5 Abs have been described in several pools of immunoglobulins and from several cohorts of either HIV-1-exposed seronegative subjects (ESN) or HIV-1-infected individuals who controlled disease progression, are named long-term non progressors (LTNP) [11]. The majority of anti-CCR5 Abs target the HIV-1 binding site at the N-terminus and the second extracellular loop (ECL2) of the receptor. Those targeting the first extracellular loop (ECL1) region induced the long-lasting internalization of the receptor and the HIV-1-blocking properties in CD4+ T lymphocytes and epithelial cells [7,12,13,14]. Importantly, the loss of anti-CCR5 Abs in some subjects was significantly associated with a progression toward disease; conversely, the LTNP status persisted in subjects who maintained anti-CCR5 Abs [15]. Therefore, CCR5 is a useful target for HIV-1 prevention. Maraviroc, a CCR5 antagonist which interferes with HIV- envelope (Env) binding to CCR5 by allosteric modulation, showed promising results in preventing HIV-1 infection in humanized RAG-hu mice and macaques. However, a 300-mg single dose was not sufficient to prevent rectal or vaginal HIV-1 transmission using an ex vivo challenge [16]. Strategies aimed at targeting CCR5, different from small molecules, were explored as alternative therapeutics, including those based on monoclonal antibodies mAbs.

A potent human IgG4 monoclonal antibody targeting CCR5, mAb004, demonstrated antiviral activity towards clade A-G HIV-1 isolates. It did not show toxicity and is currently in phase 1 clinical trial [17].

The anti-CCR5 antibody PRO 140 (Leronlimab) is a humanized monoclonal antibody that inhibits R5-tropic HIV-1 at low doses, without affecting CCR5 response to chemokines [18,19]. Several studies demonstrated a good safety profile when PRO 140 was administered once weekly by subcutaneous injection [20]. This first phase 1b trial in HIV-1 infected subjects (ISRCTN Register: ISRCTN45537485) revealed a potent, prolonged, and dose-dependent antiviral activity of PRO 140. The toxicity was minimal and a reduction in plasma viral load within 4 days from a single administration that persisted for 2–3 weeks was registered [21]. Subcutaneous PRO 140 administration demonstrated potent and prolonged antiretroviral activity with suppressed viral loads between successive doses. (NCT00642707) [22]. In a phase 2a trial, single 5-mg/kg and 10-mg/kg doses of PRO 140 were well tolerated when administered intravenously to HIV-1 infected adult subjects, showing potent and long-lived antiviral activity [23]. Dhody et al. demonstrated that HIV-1 infected individuals maintained an undetectable viral load for over 2 years in phase 2b studies in which PRO 140 was self-administered once-weekly subcutaneously [24]. A phase 2b study is evaluating the efficacy, safety, and tolerability of 350 mg weekly SQ injection of PRO 140 monotherapy for 12 weeks–maintenance of viral suppression in subjects under ART (NCT02175680).

## 3. Drugs Targeting the Lymphocyte Function-Associated Antigen-1 (LFA-1)

LFA-1 is an integrin expressed on the cell surface of lymphocytes, that functions as a cell adhesion molecule promoting migration by contact between cells or between a cell and the extracellular matrix [25]. LFA-1 is involved in the stabilization of virological synapsis allowing cell-to-cell HIV-1 transmission [26]. Upon CD4 engagement by HIV-1 Env expressed on the surface of the T donor lymphocyte, the gag precursor is recruited at the cell surface as well as HIV- 1 co-receptors and LFA-1 and its ligand ICAM-1 at the contact site, thus enabling the stabilization of the virological synapsis and allowing cell-to-cell HIV-1 transmission. The LFA-1/ICAM-1 interaction is disrupted by statins [27].

Lovastatin demonstrated anti-HIV activity in vitro by preventing LFA-1/ICAM-1 interaction, thus decreasing HIV-1 attachment to target cells [27,28,29] and promising HIV-1 inhibition and transient improvement of CD4 count in vivo [30]. A phase 2 randomized trial (LIVE study) investigated the effect of lovastatin administration at 40 mg/day during one year in HAART naïve, chronically HIV-1-infected individuals (NCT00721305). Lovostatin had no antiviral or immunomodulatory effects as HIV-1 RNA plasma load, CD4 T cell count, and expression of immune activation markers on T cells demonstrated no difference in either placebo or lovastatin groups [31,32].

Cytolin^®^ is a murine anti-human monoclonal antibody that binds to the S6F1 epitope within LFA-1, preferentially expressed by CD8^+^ T cells. It has been demonstrated that Cytolin^®^ administration to HIV-1 infected subjects reduced HIV-1 RNA and increased CD4 T cell count [33,34]. This mAb binds to both HIV-1 virions and CD8+ T cells and dendritic cells. However, it was demonstrated that it exerted anti-viral activity when bound to cells by inducing the production of an unidentified soluble factor that can inhibit HIV-1 replication [35]. The potential mechanisms of Cytolin^®^ have been investigated in an observational study on HIV-1 positive and negative subjects (NCT01048372).

## 4. Antibodies Targeting CD4-Inducible Epitopes

The binding of the HIV-1 Env glycoproteins to CD4 triggers conformational changes that allow the binding of gp120 to the chemokine coreceptor, ultimately leading to membrane fusion and virus entry [36]. Within the Env trimer, CD4-inducible (CD4i) epitopes are extremely conserved, located in and around the co-receptor binding site [37]. CD4i epitopes are commonly hidden in the Env trimer and, following CD4 engagement, the conformational change in Env results in their exposure. Some of these epitopes mapped to regions involved in the binding between Env and the chemokine receptor [38]. Therefore, antibodies targeting CD4i epitopes are interesting antiviral tools with the potential to be used in prophylactic vaccine formulations. However, the mutation ability of the virus to overcome the pressure of the induced immune response represents a major obstacle to the elicitation of such antibodies.

One of these CD4-induced conformational changes is a shift in the position of the large, surface-exposed V1/V2 variable loops of gp120, which are thought to mask the chemokine receptor-binding site on gp120 [39,40] as shown for two CD4i antibodies, named 17b and 48d. However, the whole IgG form of this mAbs did not neutralize the majority of the primary HIV-1 isolates tested, probably due to steric hindrance [40,41,42,43]. Xiang et al. identified additional three CD4i antibodies (23e, 21c, and 49e) from HIV-1-infected individuals. All of them inhibited the binding of gp120–CD4 complexes to CCR5 and neutralized laboratory-adapted HIV-1 isolates [44].

Antibodies recognizing CD4i-epitopes have both neutralizing and Fc-mediated functions, including antibody-dependent cell-mediated cytotoxicity (ADCC), phagocytosis, and trogocytosis [45,46,47].

To date, the RV144 trial is the only human vaccine trial showing efficacy in preventing HIV-1 infection, in which a protective role seems conferred by non-neutralizing antibodies. Conversely, low levels of neutralizing antibodies were developed and did not correlate with protection. This trial is based on a recombinant canarypox vector vaccine (ALVAC-HIV {vCP1521}) plus two booster injections of a recombinant glycoprotein 120 subunit vaccine (AIDSVAX B/E) (NCT00223080). The efficacy in preventing HIV-1 infection in 12,542 subjects with largely heterosexual risk in Thailand was modest but encouraging, equal to 26.2%. Vaccination did not affect the viral load or CD4+ count in subjects with HIV infection [48]. Low Env (V1V2)-specific IgA responses or a high IgG/IgA ratio combined with high ADCC responses were identified as a correlate of protection from infection [49]. Tomaras et al. showed that monomeric Env-specific IgA elicited during vaccination may modulate vaccine-induced immunity by diminishing ADCC effector function [50]. Pitisuttithum et al. demonstrated that additional boosting of the RV144 regimen improved immune responses, thus improving the efficacy of HIV-1 prevention (NCT01931358) [51]. A sieve analysis showed that breakthrough sequences from vaccinees differed from those of placebo recipients at amino acid sites 169 and 181 in V1V2, highlighting the importance of V2-specific responses [52]. This vaccine demonstrated both safety and immunogenicity in a phase 1–2a trial (NCT02404311), in which strong humoral and cellular immune responses were observed [53]. However, the ALVAC-gp120 regimen did not prevent HIV-1 infection among seronegative participants in South Africa enrolled in a phase 2b/3 trial during 24 months of follow-up (NCT02968849) [54].

## 5. CD4-Binding Site Antibodies

Some HIV-1-infected subjects developed potent broadly neutralizing antibodies (bnAbs) that are promising for their potential use in the prevention and treatment of HIV-1 infection. Specific monoclonal antibodies were isolated from those individuals and fine-tuned epitope mapping revealed the conserved epitopes were more vulnerable to neutralization. Among them, an epitope within the CD4 binding site (CD4bs) of gp120 was identified [55,56,57,58] and the first monoclonal antibody b12 was isolated [59].

Wu et al. reported the identification of three potent bnAbs, VRC01, VRC02, and VRC03, able to neutralize 90% of circulating HIV-1 strains [60]. VRC01 was further characterized and a conformationally invariant domain representing the site of initial CD4 attachment was found relevant to overcome the glycan and conformational masking, a critical point that reduced the neutralization potency of most CD4-binding-site antibodies [58].

The safety and tolerability of VRC01 were demonstrated in phase 1 clinical trials (NCT01950325; NCT01993706) [61,62]. A single infusion of mAb VRC01 significantly decreased plasma viremia and suppressed neutralization-sensitive virus strains [61]. VRC01 showed different antiviral immune functions inhibiting HIV-1 transmission and replication, as demonstrated in healthy HIV-1 uninfected individuals (NCT02165267) in which mediated antibody-dependent cellular phagocytosis was demonstrated [63]. Together with studies in adult subjects, VRC01 (along with VRC01LS and VRC07-523LS neutralizing mAbs) entered ongoing clinical studies involving the study of mother-to-child transmission of HIV-1 (NCT02256631). Preliminary data revealed that subcutaneous VRC01 as single or multiple doses is safe and well-tolerated in very young infants [64] as well as VRC01LS [65]. VRC01 efficacy was also tested in HIV-1-infected adults under antiretroviral therapy who experienced a brief analytical treatment interruption (ATI) (NCT02463227). The viral rebound was slightly delayed in these participants compared to controls, but viral suppression was not maintained by week 8 [66]. In a phase 2 trial evaluating the VRC-HIV-1MAB060-00-AB mAb administration during ATI in adults who began ART during the early phase of acute HIV-1 infection (NCT02664415), viral suppression was maintained 24 weeks after ART interruption [67]. Intravenously VRC01 administered at 8-week intervals over 20 months did not prevent overall HIV-1 acquisition in at-risk cisgender men and transgender persons in the Americas and Europe (NCT02568215) and at-risk women in sub-Saharan Africa (NCT02716675) [68]. However, further analysis revealed that HIV-1 isolates were sensitive to VRCO1 in vitro, thus suggesting that its administration was associated with a lower risk of acquisition of those isolates [68]. Together with 10E8 and PGDM1400-LS, VRC01 was incorporated in the tri-specific antibody SAR441236, which is currently under investigation in a phase 1 trial (NCT03705169).

Scheid et al. developed a new strategy to sort single B cells from a patient with high titers of bnAbs in order to overcome the challenges in antibody identification due to their high levels of somatic mutations. Through this method, the 3BNC117 mAb was isolated and identified as a more potent and broad bnAb compared to VRC01 [56]. A single 3BNC117 administration was well tolerated and demonstrated favorable pharmacokinetics. Importantly, it reduced the viral load in HIV-1-1-infected individuals by 0.8–2.5 log10 and viremia remained significantly reduced for 28 days [69]. In addition, it boosted host immunity to heterologous HIV-1 strains [70].

VRC07 is a variant of VRC01 but 5- to 8-fold more potent than VRC01, which neutralized 96% of viruses tested in vitro, including clade C [71]. VRC07-523LS is an engineered clonal variant of VRC01 in which the -LS mutation in the Fc region was designed to lengthen its half-life by increasing binding affinity to the Fc receptor [72]. VRC07-523LS administration was safe and well-tolerated in healthy adults enrolled in a phase 1, open-label, dose-escalation clinical trial (NCT03015181) at all doses (ranging from 1–40 mg/kg) and routes. In addition, it showed a promising pharmacokinetic [72].

Huang et al. isolated a CD4bs bi-specific antibody, named N6, that potently neutralized 98% of HIV-1 isolates, including those resistant to other CD4bs mAbs since it evolved to overcome the common mechanisms of resistance [73]. Preliminary data on the first phase I, open-label, dose-escalation study in healthy humans (NCT03538626) revealed its safety and tolerability with a half-life longer than 30 days (CROI).

## 6. Toll-like Receptor Agonists

Toll-like receptor (TLR) agonists are among the immune therapies under consideration for an HIV-1 cure. TLRs are a family of pattern recognition receptors (PRRs) that recognize pathogen-associated molecular patterns (PAMPs).

The ‘shock-and-kill’ strategy to eradicate the latent HIV-1 reservoir is based on latency-reversing agents (LRAs) to reactivate the provirus and subsequently enhance innate immune-mediated killing of HIV-1-expressing cells.

GS-9620 TLR-7 agonist reactivated HIV-1 in peripheral blood mononuclear cells (PBMCs) from HIV-1-infected individuals on ART therapy alone [74,75] or in combination with TLR2 agonist [76].

Toll-like receptor-3 agonist Poly-ICLC has been known to activate immune cells and induce HIV-1 replication in pre-clinical experiments. Poly-ICLC was safe and well-tolerated and therefore it could be used to induce transient innate immune as suggested by a randomized, double-blinded trial in aviremic, cART-treated HIV-1-infected subjects (NCT02071095) [77]. After 24–48 h from poly-ICLC treatment, the upregulation of innate immune pathways in PBMCs was observed at the transcriptional level, including interferon signaling and transient increases in circulating IP-10 (CXCL10) levels. These responses generally peaked by 24–48 h after the first injection and returned to baseline by day 8. No changes in both CD4+ T cell number and phenotype were registered as well as plasma viremia and effects on HIV-1 reservoirs. The authors speculated that poly-ICLC treatment is promising and higher doses might be more effective [77].

The TLR7 agonist GS-9620 (vesatolimod) induced HIV-1 expression in cells from HIV-1-infected aviremic donors on ART through a type I IFN-dependent mechanism and immune cell activation [74]. Forty-eight HIV-1 infected individuals under ART were treated with GS-9620 in a phase I dose-escalation placebo-controlled study (NCT02858401). The recently published results showed that GS-9620 was well tolerated at doses ranging from 1 to 12 mg. No differences from baseline levels in plasma HIV-1 RNA were found in the GS-9620 –treated compared to placebo-treated participants. Immune stimulation involving cytokine responses, interferon-stimulated gene expression, and lymphocyte activation were observed at dose levels above 4 mg [78].

MGN1703 is a novel Toll-like receptor 9 (TLR9) agonist, belonging to the “immune surveillance reactivators” drugs, currently being tested in a phase 3 trial for metastatic colorectal cancer [79]. It was tested in an open-label study involving 15 virologically suppressed HIV-1 infected subjects on ART receiving 60 mg MGN1703 subcutaneously twice weekly for 4 weeks (NCT02443935). The treatment resulted in increasing HIV-1 transcription and enhancing cellular immune responses through activation of plasmacytoid dendritic cells, cytotoxic NK, and CD8+ T cells. However, MGN1703 did not impact the size of persistent viral reservoirs at a cohort level [80]. In a phase 1B/2A trial MGN1703 was administered twice weekly for 24 weeks to 12 HIV-1 infected participants (NCT02443935). An increase in IFN-γ producing Gag-specific memory CD8+ T cells as well as sustained activation of memory CD4+ and CD8+ T subsets were observed. However, the measured reservoir size was not reduced and only one participant showed a time of viral rebound different from other ATI studies. Indeed, in this subject, T-cell and B-cell-mediated immunity contributed to controlling HIV-1 replication during ATI [81]. A double-blinded randomized placebo-controlled phase IIa clinical trial is ongoing to test MGN1703 in combination with two bNAbs (3BNC117 and 101074) in HIV-1-infected donors on long-term ART, the aim of the study is to investigate the reduction of the viral reservoir and the induction of the immunological HIV-1 control (NCT03837756).

## 7. Bispecific Antibodies Targeting CD4

Bispecific Abs that have two different antibody binding arms could potentially display biological characteristics better than those of any single parental antibody. They have been developed in multiple formats that combine either receptor-targeting abs or domains with anti-HIV-abs or linking the variable domains of two anti-HIV antibodies. A new generation of bnAbs with dual specificities have been engineered and demonstrated improved HIV-1 neutralization breadth and potency [82,83,84].

We focus on a combination in which at least one recognizes CD4 as a cellular target. Among them, the bispecific antibody 10E8.4/iMab, containing ibalizumab (iMab) that targets CD4, and 10E8 that targets the MPER region of HIV-1 gp41, are under investigation in a phase 1 dose-escalation study aimed at investigating the safety, tolerability, pharmacokinetics, and antiviral activity in currently recruiting HIV-1–infected and uninfected individuals (NCT03875209).

## 8. Discussion

In this review, we did not discuss the variety of immunotherapeutic approaches currently used in the field of HIV-1 cures. We focused on the cell-based methods since pre-clinical studies showed that targeting cellular molecules is a promising strategy to overcoming some limitations encountered by targeting HIV-1 targets due to the emergence of resistant viral variants. Several promising clinical trials emerged as a proof-of-concept, supporting alternative tools to develop new treatments for HIV-1 infection. Here, we underlined those approaches that are able to develop different responses on multiple fronts with the ultimate goal to provide a durable remission from the disease or the eradication of the infection. In Table 1, Table 2 and Table 3 we have provided an overview of the current clinical trials based on the selected targets discussed, with particular respect to the completed, active, and recruiting trials.

Engineered mAbs are currently used for the therapy of a broad spectrum of diseases, including cancer, autoimmune and infectious diseases [85]. Due to the key role of coreceptors in HIV-1 infection [86], we focused here on the approaches targeting the CCR5, the main coreceptor used by R5-tropic strains for entry in target cells. CCR5 mAbs offer several potential advantages compared to common therapies for their good tolerability, infrequent administration, limited interactions with other drugs, and lack of virus resistance. Pre-clinical studies on HIV-1 bnAbs showed promising results, although the first generation of bnAbs administered by passive immunization did not succeed in preventing the infection [86]. Setting up combinations of bnAbs might likely offer more encouraging results to improve the potency and the breadth of the antiviral response. In addition, a relevant breakthrough has been made thanks to promising clinical trial results from some bispecific mAbs and the development of new formats, which simplify a lot of manufacturing and physicochemical property challenges encountered by the early format. Bispecific Abs targeting cell proteins involved in HIV infection may have several advantages over other molecules. They could be engineered not only to be specific but to obtain a synergistic effect in blocking HIV infection as well, which could result in an extension of their half-life to months. This latter represents a relevant tool to develop long-acting therapies that would not have to be used every day, thus improving the quality of life and adherence of patients.

Together with neutralization, other antibody-mediated functions were characterized from naturally isolated mAbs, expanding the spectrum of targeted interventions. The new class of mAbs may also elicit an additional immune response through Fc-mediated complement-dependent cytotoxicity or Ab-dependent cellular cytotoxicity, thus improving in vivo efficacy.

Along with mAbs, TLR agonists raised interest in the field of HIV-1 cures, being already studied in cancer immunotherapies. TLR agonists offer the opportunity to both reactivate the latent virus in reservoirs and modulate the HIV-1 immune response by activating the innate immune response and priming the adaptive response. Their combination with HIV-1 bnAbs is currently under investigation as well as their role as vaccine adjuvants.

Overall, current clinical trials were not successful in decreasing the viral reservoir or delaying HIV-1 infection, and these data pointed out that the encountered challenges in defining an effective “sterilizing” therapy for HIV-1 might be overcome by using multiple approaches based on different effector functions.

## Figures and Tables

**Table 1:** Overview of monoclonal antibodies-based clinical trials.

Target	Mab	Status	Phase	ClinicalTrials.Gov Identifier
CCR5				
	PRO 140	Completed, with results	Phase 1	NCT00110591
			Phase 2	NCT00613379, NCT00642707
		Completed, without results	Phase 2	NCT02175680
			Phase 2/3	NCT02483078
		Active, not recruiting	Phase 2	NCT02355184
			Phase 2/3	NCT02859961, NCT03902522, NCT02990858
LFA-1	Cytolin	Completed	Observational	NCT010483725
CD4-i epitopes				
	RV144	Completed, with results	Phase 1	NCT03368053
			Phase 3	NCT00223080
			Follow up	NCT00337181
		Active, not recruiting	Phase 2	NCT01931358, NCT01435135
		Recruiting	Phase 1	NCT03875209
CD4-bs				
	VRC01	Completed, with results	Phase 1	NCT02471326, NCT02411539, NCT02463227, NCT02840474
			Phase 2	NCT02664415, NCT02568215, NCT02716675
		Completed, without results	Phase 1	NCT02165267, NCT02599896, NCT01993706, NCT01950325
			Phase 1/2	NCT03208231
			Phase 1/2	NCT03707977
		Recruiting	Phase 1	NCT02591420, NCT03705169
	3BNC117	Completed, with results	Phase 2	NCT02446847, NCT02588586
		Completed, without results	Phase 1	NCT03468582, NCT03254277
			Phase 2	NCT02850016
		Active, not recruiting	Phase 2	NCT03041012
		Recruiting	Phase 1	NCT04811040
			Phase 2	NCT04560569, NCT03719664, NCT03837756, NCT04319367,
			Phase 1/2	NCT04173819
	VRC07	Completed, with results	Phase 1	NCT02840474, NCT03015181
		Completed, without results	Phase 1	NCT03735849, NCT03387150, NCT03205917, NCT03803605
		Active, not recruiting	Phase 1	NCT04212091, NCT02256631, NCT03374202
			Phase 2	NCT03739996
			Phase 1/2	NCT03721510, NCT04357821
	N6	Recruiting	Phase 1	NCT03538626
			Phase 2	NCT04871113

**Table 2:** Overview of clinical trials based on antibody combinations.

Mab	Status	Phase	ClinicalTrials.Gov Identifier
VRC01+ VRC01LS	Completed, with results	Phase 1	NCT02797171
VRC01+ VRC01LS + VRC07-523LS	Active, not recruiting	Phase 1	NCT02256631
3BNC117 + 10-1074	Completed, without results	Phase 1	NCT02824536, NCT02825797
3BNC117-LS + 10-1074	Completed, without results	Phase 1	NCT03554408
3BNC117-LS + 10-1074-LS	Active, not recruiting	Phase 1	NCT04250636
3BNC117 + 10-1074 + peg-IFN-α2b	Active, not recruiting	Phase 1	NCT03588715
3BNC117 + 10-1074	Active, not recruiting	Phase 1	NCT03526848
VRC07-523LS + 10-1074 + PGT121 + PGDM1400	Completed, without results	Phase 1	NCT03928821
VRC07-523LS + 10-1074 + N-803	Recruiting	Phase 1	NCT04340596

**Table 3:** Overview of clinical trials employing TLR agonists.

Agonist	Status	Phase	ClinicalTrials.Gov Identifier
GS-9620	Completed, with results	Phase 1	NCT02858401, NCT03060447
		Phase 2	NCT02664415
	Active, not recruiting	Phase 1	NCT04364035
MGN1703	Completed, without results	Phase 1/2	NCT02443935
	Active, not recruiting	Phase 2	NCT03837756
